# Medical Education and State Medical Examinations

**Published:** 1896-08

**Authors:** William Warren Potter

**Affiliations:** Buffalo, N. Y., Member New York State Medical Examining and Licensing Board, President National Confederation State Medical Examining and Licensing Boards, 1896-'97


### MEDICAL EDUCATION AND STATE MEDICAL EXAMINATIONS.

CONDUCTED BY WILLIAM WARREN POTTER, M. D., Buffalo, N. Y.,
Member New York State Medical Examining and Licensing Board.
President National Confederation State Medical Examining and Licensing Boards, 1896-'97.

### THE STANDING OF MEDICAL COLLEGES.

THE following letter from the secretary of the Ohio State Board of Examination and Registration and the accompanying resolutions show important action :

COLUMBUS, O., June 2, 1896.

*Editor Lancet-Clinic :*

I enclose copy of resolutions adopted by the board at the last meeting in regard to the standing of medical colleges. At the last two meetings of the board there have been eight candidates examined, and of the eight the following-named gentlemen received average grades which entitle them to certificates : Chas. H. Scott, McConnellsville ; J. M. Van Tilburg, Lorain ; J. W. Kannel, Rockford ; Firmin W. Wurtsbaugh, Rush Creek ; Jas. K. Brammer, Asbury ; J. L. Kirby, Laurelville.

At the last meeting of the board a number of affidavits from graduates in medicine were passed upon and certificates were ordered to be issued, but, owing to the delay in receiving certificates from the engraver, we will not issue any before about June 10th. The applications of legal practitioners have not been taken up by the board, but most of them will be acted upon at the next regular meeting.

Very respectfully,
FRANK WINDERS,
*Secretary.*

"*Resolved,* That all medical colleges of the United States requiring a minimum of three years' study of medicine and two courses of lectures for graduation, prior to 1886, and possessing proper facilities for teaching and a faculty embracing the chairs of anatomy, physiology, chemistry, materia medica, therapeutics, medicine, surgery and obstetrics, shall be recognised as in good standing, and diplomas issued by the same, properly verified, shall entitle the holders thereof to register as graduates in medicine under the law of Ohio ; providing, that no certificate shall be issued to any applicant upon proof that his or her diploma has been obtained fraudulently or in violation of the published rules of the colleges issuing the same.

"*Resolved*, That for the ten years ending February 27, 1896, all medical colleges exacting the foregoing requirements and possessing facilities and a faculty as specified in foregoing resolution, shall, by virtue of such facts, be recognised as in good standing to and including the year 1892, but that no medical college shall be recognised as in good standing which has not since 1892 possessed the foregoing facilities and faculty, and which has not in addition exacted an entrance qualification and attendance upon three regular courses of lectures as a condition of graduation.

"*Resolved*, That on and after July 1, 1899, no medical college shall be recognised as in good standing which does not require the entrance qualification prescribed by the 'Association of American Medical Colleges' as a prerequisite for matriculation; which does not possess an adequate equipment for teaching medicine; which has not clinical and hospital facilities, based upon a minimum municipal population of 50,000, and which does not have an active faculty embracing the departments of anatomy, physiology, chemistry, therapeutics, materia medica, surgery, medicine, obstetrics, histology, pathology, bacteriology, ophthalmology, otology, gynecology, laryngology, hygiene and state medicine, and which does not enjoin attendance upon 80 per cent. of four regular courses of instruction, of not less than twenty-six weeks each, in four different years, and which does not exact an average grade of 75 per cent. on examination as conditions of graduation; providing that the rule relative to population as a basis for clinical and hospital facilities shall not apply to institutions under state control, and that, by virtue of such control, receives, gratuitously, patients from all parts of the state in which such colleges are located."

---

THE Ohio State Board of Medical Examiners did a good thing when it passed a rule by which it regards as irregular any medical college located in a city of less than 50,000 inhabitants. One so-called school of medicine in Ohio promptly closed shop on the promulgation of the above order. The State Board is very apt to do a whole lot of good right straight along, for its destiny is to get directly in the way of such things.

It was very gratifying to all concerned to observe the way in which the Ohio State Medical Society unanimously endorsed every bit of the action of its legislative committee. A prominent quack who stood by and looked on didn't smile, but looked as though he felt the seriousness of his situation. His occupation has slipped out of his hands, and it is well that he should engage in another business or go back to Germany, where he says his school flourishes. It will be hard to endure. America is a splendid burden-bearer, so it may be said his absence can be borne. Where is that part of a gentleman's wardrobe commonly called a handkerchief ?—*Lancet-Clinic.*

---

### DIVISION OF MEDICAL EXAMINATIONS.

THE following is the report of the committee on division of state medical examinations :

*To the President of the State Board of Medical Examiners, representing the Medical Society of the State of New York :*

In compliance with the instruction of the New York State Medical Examining and Licensing Board, made at its last annual meeting, as well as the published notice from the regents' office, the undersigned committee met at the Fifth Avenue Hotel, New York City, February 15, 1896, to listen to arguments pro and con relating to the division of medical examinations for license to practise in this state.

After listening to the various arguments submitted at the hearing, and duly weighing the same, your committee begs to submit the following report :

*First*—The committee was addressed in favor of the division by the following-named gentlemen : Profs. J. H. Raymond and W. W. Browning, on behalf of Long Island College Hospital ; Profs. R. A. Witthaus and W. Gilman Thompson, on behalf of the Medical Department University of the City of New York ; Prof. Phœbe J. B. Waite, on behalf of the New York Medical College and Hospital for Women (Homeopathic) ; Prof. George W. Boskowitz, on behalf of the New York Eclectic Medical College.

*Second*—These arguments may be briefly summarised as follows : According to Dr. RAYMOND, a student has now to pass in the colleges an examination on about twenty-three subjects, whereas there were formerly but seven ; that it is his belief, as a result of his experience, if the men were relieved of anxiety by passing the state examination at the end of two years in the so-called elementary or fundamental subjects—anatomy, chemistry and physiology—it would leave the last year in college to be devoted to the practical branches—surgery, obstetrics, practice, and the like—thus insuring a more perfect equipment in the latter; that better clinical instruction could be given in the final year if the state examinations were so divided; that Saturdays and holidays were now spent in preparing for the state examination instead of in the clinic rooms; that as academic institutions divided their examinations, why should not the state? that medical colleges have adopted this division of examinations, hence the state ought to conform to their practice; that, finally, the division is urged in the interest of impecunious students whose existence may depend upon passing the state examinations. Dr. Raymond admitted the good work that the state board was accomplishing, and said his college would hail the day when the law was changed to make a four years' course obligatory.

Dr. Browning thought that a state examination on the elementary subjects one year prior to graduation would be a better test, than an examination given at the close of the college course to demonstrate that a man had sufficient knowledge of anatomy to be admitted to practice; that, adopting the theory that an easy examination in anatomy is given at the close of the college course rather than a severe one a year before license is granted, then the present syllabus should be withdrawn; and that in case it is deemed advisable to divide the examinations, legislation will become necessary to permit it.

Dr. Witthaus claimed the real question at issue to be whether a graded system of instruction is or is not better than the old-fashioned one of going over the same thing two or three times; that he gets better results in chemistry under the present system, as students study harder and learn more; that he would not have the severity of the chemistry examinations abated one particle; that it is of the first importance in the advances that medicine is making that a physician should be thoroughly grounded in the fundamental branches before going on with the others; that he would not have the slightest abatement in the severity of those examinations; and, finally, that it is not within the power of the board to make this change, hence legislation will become necessary to permit it.

Dr. Thompson affirmed that the present working of the state examinations, conducted as they are at one time, is a hindrance to the graded course system; that a graded state examination could be conducted on severer lines than is done under the present plan, which latter idea he would favor; that we are apt to attach too much importance to examinations, but they are tests that we must apply, and this can be done with greater advantage in a graded system; that the present method of conducting state examinations is an interference with the graded method, and that he fully concurs in the remarks made by Drs. Raymond and Witthaus.

Dr. Waite favored the proposed change in state examinations because it conformed to the college graded system, relieved the students of mental strain and otherwise favored advanced medical education.

Dr. Boskowitz favored the division of the state examination, but advanced no arguments others than those put forth.

*Third*—The committee was addressed in opposition to the division by Prof. James W. McLane, on behalf of the College of Physicians and Surgeons of New York, and Dr. Daniel Lewis, president of the State Board of Health.

*Fourth*—These arguments may be epitomised as follows: Dr. McLane asseverated that the function of the college is one thing and that of the state quite another; that the first function of the state is to exclude unqualified men from entering upon the study of medicine; that all found qualified are turned over to a medical school to be educated in medicine; that the college is educating the man, and if it judges him to be competent it grants him a diploma; that the state now inspects him, and if found qualified it licenses him to practise medicine; that the examining board is not an educational body but a licensing body; that if a division is made, many men will be examined unnecessarily, because many men after the first or second year drop out and do not come up for final examination; that he doubts if there are many excluded by the state examinations who ought to enter the practice of medicine; that if incompetent persons slip through the schools they ought to be caught in the state net; that the object of this law was to raise the standard of medical education; that this movement is not in the direction of raising the standard; that the argument that the examination as at present conducted interferes with a graded course is not tenable; that the colleges may pursue the course they find best, but the state is to decide whether a man is competent to practise, which can only be determined by final examination; that the law, as at present administered, is working well; that it has stimulated the schools to teach the men well, as each feels a personal pride in having their graduates pass the state board; and, finally, that it is better for all imperfectly equipped men in elementary branches to be excluded from the profession of medicine.

Dr. Lewis stated that he had been familiar with the medical practice laws from their inception; that Prof. McLane had correctly interpreted the motives of those who had drafted the statutes, wherein a licensing body and not an educational one was aimed at—one that should test the qualifications of a man for the practice of medicine after graduation; that it was desired to make it simple and effective, complications being avoided; that the fact that it has been successful down to the present time is a potent reason why it should not be modified; that efforts are made annually to annul its provisions that have only been thwarted by resisting all proposed amendments; that it is not safe to undertake the modification of a satisfactory law ; that it would be impossible to make the proposed division, or graded examination, apply to graduates from the other states and countries ; that it could not be made to apply to those going out of the state to practise ; that complications in making this change would be almost endless, and that the general profession is satisfied with the present law and the manner of its execution. Dr. Lewis was of the opinion that a change could be made in the direction named, should it be deemed advisable, without further legislation.

*Fifth*—Communications were read from Dr. John Cronyn, Buffalo, president of Niagara University, and others, but these did not advance any arguments not already presented by the speakers who addressed the committee.

CONCLUSIONS.

Your committee, after mature deliberation and a careful consideration of all the arguments presented, believes that it would be unwise to make any change or modification in the present methods ef conducting the state examination for license to practise medicine for the following reasons :

1. The operations of the law are well understood as at present administered, both by the colleges and the students. It has taken all this time to get the machinery in thorough working order and to familiarise all concerned with its duties and requirements. To disrupt its operations by such a radical change as is proposed would be prejudicial to the best interests of higher medical education.

2. The majority of the colleges in the state, as well as physicians who are not teachers, appear to be opposed to such a change ; or, to state this in another way, out of twelve undergraduate medical colleges but four advocate a change either in person or by letter. Moreover, so far as our knowledge extends, the entire profession, independent of college teachers, is not in favor of any change.

3. The functions of the state may be exercised to prevent unqualified men—namely, those deficient in preliminaries—from beginning the study of medicine, and it also may exercise its functions to prevent improperly qualified graduates from practising medicine by withholding its license after due examination, but it cannot properly exercise any control over the student between his entrance examination and his examination for state license. In other words, it is unlawful, if not unconstitutional, for the state to interfere with a student after he has entered college until he finally comes up for state license. In still other phraseology, the state, may determine the preliminaries, but its medical examination is a post-graduate and not an under-graduate test.

4. Finally, your committee believes that in the interest of simplicity, fairness and higher medical education, the present method should prevail. We are in great doubt as to the legality of dividing the examinations without amending the law, and we think it would not be prudent to invoke legislation on the subject.

Respectfully submitted,

GEORGE R. FOWLER, M. D.
WILLIAM WARREN POTTER, M. D.
MAURICE J. LEWI, M. D.

